# Homœpathy, Allopathy, and “Young Physic”

**Published:** 1846-04

**Authors:** 


### PART II.—REVIEW.

### ARTICLE III.

*Homœopathy*, *Allopathy*, *and* "*Young Physic.*" By John Forbes, M. D., F. R. S., Editor of the British and Foreign Medical Review, etc. etc. Philadelphia: Lindsay and Blakiston. 1846. pp. 124. (From the Publishers.)

This work is a reprint, in a separate form, of an article of the above named Review, and is marked by the clearness and vigor characteristic of the style of the editor, and by a liberality, fairness, and indulgence towards the Homœopathic doctrines, carried, we think, even to excess. Of this, however, our readers shall be the judges, as we lay before them an analysis of the contents of the book.

Samuel Hahnemann, the founder of the system, was born at Misna, in Upper Saxony, in the year 1755, and died at Paris only three years since, in the eighty-eighth year of his age. "No careful observer of his actions or candid reader of his writings, can hesitate for a moment to admit that he was a very extraordinary man—one whose name will descend to posterity as the exclusive excogitator and founder of an original system of Medicine, as ingenious as many that preceded it, and destined, probably, to be the remote, if not the immediate cause of more important fundamental changes in the practice of the healing art, than there have resulted from any, promulgated since the days of Galen himself."* He is further described as a man of genius, learning, great industry, and energy, deserving of being ranked among the greatest systematists and theorists. In reference to the character of Hahnemann, to judge from his writings, it appears singularly enthusiastic, sincere, upright, and honorable; we see no reason to doubt his good faith. He was the object of unjust persecution, obliged to take refuge in a foreign country and deserves, for that reason alone, to be well treated by mankind. But we are not able to perceive by what title he is to be ranked among the great theorists of medicine. As a *theorist* his views have always appeared to us whimsical and absurd in the highest degree. That we are not influenced in this opinion by prejudice, may be inferred from the following passage from the preface to the American edition of the Organon, written by one of the leading advocates of the system. "For myself I am generally considered as a disciple and adherent of Hahnemann, and I do indeed declare I am among the most enthusiastic in doing homage to his greatness, but, nevertheless, I declare, also, that since my first acquaintance with homœopathy, down to the present day, I have never yet ac-

* Page 7.

cepted a single theory of the Organon, as it is there promulgated."*

The first idea of the system is said to have found its way into the head of the author in the following manner. Having occasion, about the year 1790, to experiment upon himself with cinchona, he thought he discovered that it possessed the power of exciting symptoms like those of ague, hence arose in his mind the dawn of more rational therapeutics, "may not the power of the bark to cure ague," said he, "depend upon its power to excite in the healthy body a similar disease." He then proceeded to try the effects of other remedies on himself, and always, it is said, with the same result. Examining then the records of medicine in reference to the effects of poisons and strong drugs, and finding everything, as he believed, confirmatory of his views, he hesitated no longer but "promulgated the grand and universal law, that every (dynamic) disease is best cured by that medicine which is capable of producing, in the healthy body, similar symptoms, or a similar disease, or as it is stated more briefly, *similia similibus curantur*, like are cured by like, i. e.: homœopathically."

This is the foundation of his system, and in order to cure a disease according to it, it is only necessary to find a medicine capable of exciting in the healthy body, symptoms nearly the same as those noticed in the disease. He gave as the *rationale* of the cures thus effected the following, viz: that of two similar actions developed in the same part, the stronger destroys the weaker.

The principle and explanation being thus given, we come to the method of administering the remedies. This is done in *infinitesimal* doses, or in quantities so small as to be entirely inappreciable to the senses. This does not appear to be necessary to the homœopathic principle, but was adopted because the practitioner should only desire to excite a *medicinal disease*, slightly greater than the natural one, so that when the latter has been once cured, but little trace of the former should remain. It appears, that for this purpose the attenuation of the medicine can scarcely be carried too far if it be well chosen. "It is of little consequence that this attenuation may go so far as to appear impossible to common physicians, whose minds are only conversant with gross material notions." Although this attenuation of the dose did not necessarily result from the homœopathic principle, yet it has now been so universally adopted, as to be considered an essential part of the practice."

Nor is this to be wondered at, for if in acute diseases we administer medicines in active doses upon that principle, the results may easily be supposed less favorable, than when

* Preface to Second American Edition, page 15.

these are given in such quantities as to allow the free action of the *vis medicatrix naturæ.*

The efficacy of doses infinitely small, is, however, accounted for by Hahnemann by the increased sensibility of the diseased part. The following are the doses used:

1st. One hundredth of a drop or grain.
2d. " ten thousandth do do
3d. " millionth do do and so on to the thirtieth, which contains one decillionth of a drop or grain. In order to aid the imagination in partly comprehending calculations, so entirely beyond the reach of the minds of men, the following observations are given from the work of Dr. Alexander Wood. That they may be understood, it must be recollected, that at the 6th dilution the dose is the billionth of a grain or drop. "A billion of moments have not elapsed since the (mosaic) creation of the world, and to produce a decillionth, that number, must be multiplied by a million seven separate times. The distance between the earth and the sun is 95 millions of miles; twenty of the homœopathic globules, laid side by side, extend to about one inch, so that 158,400,000,000 of such globules would reach from the earth to the sun. But when the thirtieth dilution is produced, each grain is divided into 100,000,000,000,000,000,000,000,000,000,000,000,000,000,000,000,000,000,000,000,000,000 parts, so that a single grain of any substance in the thirtieth dilution, would extend between the earth and the sun 1,262,626,262,626,262,626,262,626,262,626,262,626,262,626,262,626,262 separate times."

It is to be remarked that the activity of these small doses depends upon the number of times it has been shaken, or the length of time it has been rubbed, each shake adding to its power, so that, says Hahnemann, "experience has forced me to reduce to two, the number of agitations, of which formerly I ordered ten at each dilution."

As many articles of diet or drink are used in such quantities as to operate sensibly upon the system, it becomes necessary, during the use of these remedies, to confine the patient to a low regimen, and a long list is given of articles prohibited and things to be avoided, such as coffee, tea, beer, wine, spirits, spices, aromatics, &c., also over indulgence, sedentary life, exciting books, sexual indulgence, anger, vexation, scorn, &c.

"So far it must be allowed," says one author, "the doctrines of Hahnemann have either a show of reason in themselves, or claim to be founded upon grounds even superior to reason—experience and experiment." But we now come to a part which must at present at least appear to be the work of pure imagination. He maintains that all chronic diseases, with scarcely an exception, are the result of *syphilis*, *sycosis*, or *psora*, common itch. This latter he considers to be the origin of seven-eighths of all the chronic diseases and to be only capable of being cured by certain articles called anti-psoric.

This is a brief and imperfect, but yet as far as it goes, a correct view of the principles and theories of homœopathy. In order, however, to understand it more fully it must be known that the followers of this system attach but little, if any, importance to the morbid changes of the fluids or organs, or what, in ordinary language, is termed pathology. Disease is looked upon as spiritual, and the operation of medicines regarded in the same light. "The vital principle, as a spiritual dynamis, connot otherwise be assailed and affected than in a (dynamic) spiritual manner, neither can such morbid disturbances, or in other words, such diseases, be removed by the physician, except in like manner, by means of the spiritual (dynamic virtual) countervailing agency of the suitable medicines acting upon the same vital principle."*

Dr. Forbes (whose arrangement we mostly follow, giving but an abstract of his views,) then proceeds to examine into its truth.

1st. Is it true that the cinchona is capable of producing an ague, or something nearly resembling it? Few will hesitate to pronounce that it possesses for mankind at large, no power of the kind, and the same is true of most homœopathic remedies in common use. The symptoms ascribed to these, and of which there are several hundred for one single article, are either imaginary, accidental, or have no resemblance to those of the disease they are employed to cure. "By what means," says Hahnemann, "do they drown the distant roar of the enemy's cannon which carries terror into the breast of the soldier? By the deep-mouthed clangor of the big drum."† And a number of illustrations equally applicable are adduced.

2d. Is it true that substances, inert in large doses, acquire a wonderful activity when reduced to quantities beyond the power of imagination distinctly to conceive? "To be called upon to believe that the decillionth of a grain of oyster shell or charcoal is capable of producing hundreds of the most formidable symptoms, and of curing, as by magic, the most inveterate diseases, while we take ounces, nay pounds, of the same substances into our stomachs, with no other convenience than its mechanical bulk, seems so gratuitous an outrage on human reason, that the mind recoils instinctively from the proposition."

We pass over a number of theoretical points as the psoric-origin of most chronic diseases, &c., neither shall we spend

* Hahnemann. Organon, p. 59.

† Op. Cit. p. 89.

time in bringing forward objections or arguments against it. This were a waste of time, nor should we have devoted to it even this attention, had it not been that its claims, as a practical system, are less known than are its doctrines among the medical profession. Here again, we think that Dr. Forbes has made his representations of its results more favorable than justice would strictly warrant. It is no doubt true that "the disciples of Hahnemann are spread over the whole world." That "there is not a town of any considerable size in Germany, France, Italy, England or America, that does not boast of possessing one or more homœopathic physicians, not a few of whom are men of huge respectability and learning." It may also be true that "the Professor of Pathology in the university of Edinburgh has become a convert to its doctrines." But it is not the less true that the methods of cure recommended in its standard works, and the cases reported by its advocates, are altogether worthy of the doctrines. Take, as an example the following from the Organon, page 14. Speaking of affections of the stomach with acrid eructations, he says "these gastric affections of dynamic origin are commonly produced by a disturbed state of the mind, (grief, freight, anger,) cold, exertion of the mind or body immediately after eating, and sometimes even after a temperate enjoyment of food. Neither the tratrate of Antimony nor Ipecacuanha are suited to the purpose of removing this dynamic aberration." "But if the patient should only smell once to a globule of sugar of the size of a mustard seed, impregnated with the thirtieth dilution of pulsatilla, he is then cured in the space of two hours."

The question, however, may be considered in a different point of view, and putting aside the absurdity of the supposition that such means are capable of affecting the system, what are the evidences that cures are effected by it. On this point there is absolutely no means of judging at present. To afford sufficient data for this purpose, it would be necessary, that of a great number of patients placed in similar hygenic circumstances, a part should be treated "homœopathically, and the others be treated apparently in the same manner but with fictitious globules." Experiments of this kind, conducted on a large scale, can only determine the absolute potency or impotency of this plan of treatment, for whatever number of persons recover under its application, there is no means of knowing whether the same result would not have taken place without any medical treatment.

There are, however, at the present time, numbers of cases reported, treated homœopathically as well as otherwise, from which some judgment may be formed of the comparative value of this with other varieties of treatment. Among these, the most reliable part is the report of cases treated at the hospital of the Sisters of Charity, which was opened in Vienna, in 1832, and since 1835 has been under the care of Dr. Fleischmann, who treats all patients homœopathically. From the beginning of 1835 to the end of 1843, there were treated 6551 patients, with the following results:

| | |
|---|---|
| Remaining from 1834, - - | 27 |
| Admitted, - - - - | 6524 |
| Cured, - - - - | 5980 |
| Dismissed uncured, - - - | 112 |
| Died, - - - - | 407 |
| Remaining, - - - - | 50 |

The following extract will show the result in some of the more important chronic and acute diseases.

| | Admit'd. | Cured. | Uncured. | Died. |
|---|---|---|---|---|
| Abscess of the Brain, | 3 | | | 3 |
| Cancer of Stomach and Uterus, | 5 | | 2 | 3 |
| Ascites, | 14 | 10 | 1 | 3 |
| Fever, excluding Typhus, | 1036 | 1007 | 1 | 17 |
| Typhus Abdominalis, | 819 | 669 | 2 | 140 |
| Pneumonia, | 300 | 280 | | 19 |
| Bronchitis, | 15 | 15 | | |
| Pleuritis, | 224 | 221 | | 3 |
| Phthisis, | 98 | | 27 | 71 |

It will readily occur to our readers that such tables, destitute of classification, date of admission, length of time required for cure, age, sex, symptoms by which each disease was recognized, are destitute of value as regards positive and accurate results. There are circumstances, too, which render the statements still more doubtful, such, for example, as the disproportion between the number of cases of bronchitis and those of pneumonia. But waiving all objections, and taking the cases as of average severity, what are the results? Certainly not more favorable than would be found with the ordinary method of practice. It would be useless to institute a close comparison between these results and those obtained elsewhere, under different modes of treatment, and in circumstances quite different. But it is seen at a glance, that of the diseases usually accounted incurable, as Cancer, Abscess of the Brain, and Phthisis, all the cases are either fatal or dismissed uncured, which is usually the same.

Of acute diseases, as Pneumonia, &c., the ratio of mortality is nearly the same as that usually observed under the ordinary modes of treatment. So that Dr. Forbes is disposed to admit, upon the authority of the table, of which the above is a small part, that the results are nearly the same as are found in ordinary practice. We would here add a word in regard to other trials which have been made of this practice.

It is known that in 1835, Andral administered homœopathic medicines at La Pitié to a large number of patients without sensible effects. At St. Petersburg, the Medical Council after having tried, declared it as either useless or dangerous in cases where active treatment is required. At Naples, the public authorities revoked, after a trial of forty-five days, the permission which had been given for the establishment of a clinic of homœopathy. At Paris, besides those of Andral, trial was made of it in the words of M. Bailly, at the Hotel Dieu, with medicines prepared in Germany at the same establishment where Hahnemann procured his supplies. They were without effect, and the homœopath who directed them, retired after four or five months trial. At Lyons, in 1830, Dr. Pointe, professor of Clinical Medicine at the Hotel Dieu, put at the disposition of Dr. Gueyard, thirty beds. This latter, in presence of several physicians and pupils, examined the patients, prescribed the medicines, regimen, &c., and after 17 days of trial he gave it up.*

Dr. Forbes, after the report of Dr. Fleischmann, passes in review the cases given by Dr. Henderson in a work entitled "An enquiry into the Homœopathic practice of Medicine." We will not follow him in this part of his work. It is sufficient to give his conclusion, "that the amount of success obtained by Dr. Henderson in the treatment of his cases, would have been considered by ourselves very satisfactory, had we been treating the same cases according to the rules of ordinary medicine."

"In making these admissions in respect to the instances of treatment supplied by Drs. Henderson and Fleischmann, we wish formally to guard ourselves against being supposed to admit" "that the result of the homœopathic treatment generally, is as successful as the ordinary treatment generally." "On the other hand, we have not a little positive evidence to prove that it has often failed to cure in cases where, according to its principles and the alleged experience of its professors, it ought to have cured, and in which allopathy did effect a cure."

"The guiding principle of homœopathy appears to us to be of that character which must render its exercise very injurious to medicine as a branch of science. Based, as it is, on mere extrinsic, secondary phenomena, or symptoms, and exclusively engaged in the search for and adaptation of specific remedies to such phenomena, we cannot but regard it as calculated to destroy all scientific progress in medicine, and to degrade the minds of those who practice it." With such views of homœopathy, how are to be explained the cures effected under its ministration. Our author, in common with all judicious and observing men, attributes them to the power of nature, a power which he seems to think is greatly overlooked and undervalued by medical men of the present day.

* Dict. de Medicine, vol. xv, p. 358.

He even goes into a lengthened argument to show that nature can cure diseases without the interference of art. For ourselves we do not think this necessary, since every observing physician knows that, not only among savages, but even among civilized men, a great majority of all the diseases which occur, are not subjected to any medical treatment. Pneumonia, Pleuritis, Bronchitis, and all the more serious diseases, occur daily among the laboring classes, and especially those at a distance from physicians, and get well without medical aid. Many of these have been treated with simple diet and drinks, and found to terminate as favorable as under ordinary treatment, witness Typhoid Fever, Phthisis, etc. In this point of view, he thinks homœopathy likely to be of immense advantage to medicine, by enabling us to judge of the power of nature to cure diseases, and of their course when not interfered with by art.

We cherish no such expectations of the benefits to be derived from homœopathy. All makers of systems of late, if not always, have retarded medical science, or caused it to retrograde. Witness Broussais. Nor can it be otherwise at present. The time has not arrived for completing the edifice, but only for patiently gathering suitable materials.

If these doctrines are so absurd as is shown by Dr. Forbes, can any good be expected to result from them? If mankind are better without medicine, were it not wiser to say so at once, rather than abuse them with doses incapable of having any effect. If there were advantage to be gained from such a course, the profession might have reaped it long ago, for it was long since stated that "medicine is the art of amusing the patient while nature cures the disease."

The humblest physician who employs, in curing diseases, all the resources within his reach, we respect. But if he chooses to desert the only track which can lead to a full knowledge of the human system and its morbid states, and range himself under the banner of homœopathy, hydropathy, botanic, &c., or advocate the virtues of a "panacea" elixir of life, and other preparations of the same kind, we look upon his efforts as lost to science and humanity, however advantageous they may be to himself. It was this view, we presume, which influenced the Supreme Court of the State of New York in deciding homœopathy to be quackery. Far from expecting that it will be of service to science or mankind, we see clearly that it can only obstruct the progress of the former, that valuable lives will be, as they have been, sacrificed by hundreds and thousands upon the shrine of a false theory, and to the love of gain. Nor is this consideration the less painful from the fact that other systems have also had their victims. That antimony and calomel, and violent medicines of all sorts, have been used from habit, routine, and ignorance, until they also have destroyed life and health. It is impossible, in many parts of our own country, not to see that it is but one error following another; that homœopathy is but a reaction against the excessive use of drugs. And this leads us to the essential part of the work we are reviewing, the consideration of the present state of medical practice, and the reforms which are necessary. It is for the purpose of laying these before our readers, as well as in part to make them acquainted with the progress of various medical doctrines, that we have noticed this work so much in extenso. We will only add that the difficulties at the present time, do not, in our own judgment, rest so much with the science, as the modes in which it is taught and practiced. The following is the concluding part of the work to which we referred.

"It would be presumptuous in us, in the present stage of the question, to attempt to give even a formal outline or sketch of the Reform in Practical Therapeutics which appears so necessary, and which we believe to be impending. This is a work which can only be the result of mature reflection, and of the labor of many years and many hands. All which we can think of attempting at present, is to set down, almost at random, a few of the various considerations that press upon us, touching the many things to be thought of and done, the manifold evils to be abated, the manifold benefits to be achieved, by the enthusiastic and active spirits whom we have heretofore sportively personified under the name of 'Young Physic,' and to whom we look with confidence for the consummation of the great Reformation which assuredly will come.

"In submitting these suggestions to criticism, we would request, that their extemporaneous and undigested character be borne in mind. All of them, we believe, to be true and just; many of them of high importance; and although more mature reflection may prove some of them, at least, to be neither the one nor the other, we shall, nevertheless, not regret having written them. They may excite others to consider this momentous subject, and thus elicit from better minds, thoughts worthier of remembrance and fruitful of greater things.

### Cogitanda—Excogitanda—Agenda.

"1. To endeavor to ascertain, much more precisely than has been done hitherto, the natural course and event of diseases, when uninterrupted by artificial interference; in other words, to attempt to establish a true Natural History of human diseases.

"2. To reconsider and study afresh the physiological and curative effects of all therapeutic agents, with a view to obtain more positive results than we now possess.

"3. To endeavor to establish, as far as is practicable, what diseases are curable and what are not; what are capable of receiving benefit from medical treatment and what are not; what treatment is the best, the safest, the most agreeable; when it is proper to administer medicine, and when to refrain from administering it; &c. &c.

"4. To endeavor to introduce a more philosophical and accurate view of the relations of remedies to the animal economy and to diseases, so as to dissociate in the minds of practitioners the notions of *post hoc* and *propter hoc.*

"The general adoption by practitioners in recording their experience, of the system known by the name of the *Numerical Method*, is essential to the attainment of the ends proposed in the preceding paragraphs, as well as in many that are to follow.

"5. To endeavor to banish from the treatment of acute and dangerous diseases, at least, the ancient axiom, *melius anceps remedium quam nullum*, and to substitute in its place the safer and wiser dogma—that where we are not certain of an indication, we should give nature the best chance of doing the work herself, by leaving her operations undisturbed by those of art.

"6. To endeavor to substitute for the monstrous system of Polypharmacy now universally prevalent, one that is, at least, vastly more simple, more intelligible, more agreeable, and, it may be hoped, one more rational, more scientific, more certain, and more beneficial.

"7. To direct redoubled attention to hygiene, public and private, with the view of preventing diseases on the large scale, and individually in our sphere of practice. Here the surest and most glorious triumphs of medical science are achieving and to be achieved.

"8. To inculcate generally a milder and less energetic mode of practice, both in acute and chronic diseases; to encourage the Expectant preferably to the Heroic system—at least where the indications of treatment are not manifest.

"9. To discountenance all active and powerful medication in the acute exanthemata and fevers of specific type, as small-pox, measles, scarlatina, typhus, &c., until we obtain some evidence that the course of these diseases can be beneficially modified by remedies.

"10. To discountenance, as much as possible, and eschew the habitual use (without any sufficient reason) of certain powerful medicines in large doses, in a multitude of different diseases, a practice now generally prevalent and fraught with the most baneful consequences.

"This is one of the besetting sins of English practice, and originates partly in false theory, and partly in the desire to see manifest and strong effects resulting from the action of medicines. Mercury, iodine, colchicum, antimony, also purgatives in general and blood-letting, are frightfully misused in this manner.

"11. To encourage the administration of simple, feeble, or altogether powerless, non-perturbing medicines, in all cases in which drugs are prescribed *pro forma*, for the satisfaction of the patient's mind, and not with the view of producing any direct remedial effect.

"One would hardly think such a caution necessary, were it not that every-day observation proves it to be so. The system of giving and also of *taking* drugs capable of producing some obvious effect—on the sensations, at least, if not on the functions—has become so inveterate in this country, that even our *placebos* have, in the hands of our modern doctors, lost their original quality of harmlessness, and often please their very patients more by being made unpleasant!

"12. To make every effort not merely to destroy the prevalent system of giving a vast quantity and variety of unnecessary and useless drugs, (to say the least of them,) but to encourage extreme simplicity in the prescription of medicines that seem to be requisite.

"Our system is here greatly and radically wrong. Our officinal formulæ are already most absurdly and mischievously complex, and our fashion is to double and redouble the existing complexities. This system is a most serious impediment in the way of ascertaining the precise and peculiar powers (if any) of the individual drugs, and thus interferes, in the most important manner, with the progress of therapeutics.

"We are aware of the arguments that are adduced in defence of medicinal combinations. We do not deny that some of these combinations are beneficial, and therefore proper; but there cannot be a question as to the enormous evils, speaking generally, resulting from them. Nothing has a greater tendency to dissociate practical medicine from science, and to stamp it as *a trade*, than this system of pharmaceutical artifice. It takes some years of the student's life to learn the very things which are to block up his path to future knowledge. A very elegant prescriber is seldom a good physician. And no wonder. Tailors, barbers, and dancing-masters, however learned they may be in the externals of gentility, are not expected to be fine gentlemen or men of fashion.

"13. To endeavor to break through the routine habit, universally prevalent, of prescribing certain determinate remedies for certain determinate diseases or symptoms of diseases, merely because the prescriber has been taught to do so, and on no better grounds than conventional tradition.

"Even when the medicines so prescribed are innocuous, the routine proceeding impedes real knowledge by satisfying the mind, and thus producing inaction. When the drugs are potent, the crime of mischief-making is superadded to the folly of empiricism. In illustration, we need merely notice the usual reference, in this country, of almost all chronic diseases accompanied with derangement of the intestinal functions, to 'affection of the liver,' and the consequent prescription of *mercury* in some of its forms. We do not hesitate to say, that this theory is as far wrong as the practice founded on it is injurious; we can hardly further enhance the amount of its divarication from the truth.

"14. To place in a more prominent point of view the great value and importance of what may be termed the physiological, hygienic, or natural system of curing diseases, especially chronic diseases, in contradistinction to the pharmaceutical or empirical drug-plan generally prevalent. This system, founded as it is on a more comprehensive inquiry into *all* the remote and exciting cause of disease, and on a more thorough appreciation of *all* the discoverable disorders existing in all the organs and functions of the body, does not, of course, exclude the use of drugs, but regards them (generally speaking) as subservient to hygienic, regimenal, and external means,—such as the rigid regulation of the diet, the temperature and purity of the air, clothing, the mental and bodily exercise, &c., baths, friction, change of air, travelling, change of occupation, &c. &c.

"15. To endeavor to introduce a more comprehensive and philosophical system of Nosology, at least in chronic diseases, whereby the practitioner may be led less to consider the name of a disease, or some one symptom or some one local affection in a disease, than the disease itself—that is, *the whole* of the derangements existing in the body, and which it is his object to remove, if possible.

"16. To teach teachers to teach the rising generation of medical men, that it is infinitely more *practical* to be master of the elements of medical science, and to know diseases thoroughly, than to know by rote a farrago of receipts, or to be aware that certain doctors, of old or of recent times, have said that certain medicines are good for certain diseases.

"17. Also to teach students that no systematic or theoretical classification of diseases, or of therapeutic agents ever yet promulgated, is true, or anything like the truth, and that none can be adopted as a safe guide in practice. It is, however, well that these systems should be known; as most of them involve some pathological truths, and have left some practical good behind them.

"18. To endeavor to enlighten the public as to the actual powers of medicines, with a view to reconciling them to simpler and milder plans of treatment. To teach them the great importance of having their diseases treated in their earliest stages, in order to obtain a speedy and efficient cure; and, by some modification in the relations between the patient and practitioner, to encourage and facilitate this early application for relief.

"19. To endeavor to abolish the system of medical practitioners being paid by the amount of medicine sent in to their patients; and even the practice of keeping and preparing medicines in their own houses.

"Were a proper system introduced for securing a good education to chemists and druggists, and for examining and licensing them—all of easy adoption—there could be no necessity for continuing even the latter practice; while the former is one so degrading to the medical character, and so frightfully injurious to medicine in a thousand ways, that it ought to be abolished forthwith, utterly and for ever.

"20. Lastly, and above all, to bring up the medical mind to the standard necessary for studying, comprehending, appreciating, and exercising the most complex and difficult of the arts that are based on a scientific foundation,—the art of Practical Medicine. And this can only be done by elevating, in a tenfold degree, the preliminary and fundamental education of the Medical practitioner." D. B.

